# Support Systems of Clinical Decisions in the Triage of the Emergency Department Using Artificial Intelligence: The Efficiency to Support Triage

**DOI:** 10.15388/Amed.2023.30.1.2

**Published:** 2023-01-24

**Authors:** Eleni Karlafti, Athanasios Anagnostis, Theodora Simou, Angeliki Sevasti Kollatou, Daniel Paramythiotis, Georgia Kaiafa, Triantafyllos Didaggelos, Christos Savvopoulos, Varvara Fyntanidou

**Affiliations:** Emergency Department, AHEPA University General Hospital of Thessaloniki, 55636, Thessaloniki, Greece; First Propaedeutic Internal Medicine Department, AHEPA University General Hospital of Thessaloniki, 55636, Thessaloniki, Greece; Advanced Insights, Artificial Intelligence Solutions, Ipsilantou 10, Panorama, 55236 Thessaloniki, Greece; First Propaedeutic Internal Medicine Department, AHEPA University General Hospital of Thessaloniki, 55636, Thessaloniki, Greece; First Propaedeutic Internal Medicine Department, AHEPA University General Hospital of Thessaloniki, 55636, Thessaloniki, Greece; First Propaedeutic Surgery Department, AHEPA University General Hospital of Thessaloniki, 55636 Thessaloniki, Greece; First Propaedeutic Internal Medicine Department, AHEPA University General Hospital of Thessaloniki, 55636, Thessaloniki, Greece; First Propaedeutic Internal Medicine Department, AHEPA University General Hospital of Thessaloniki, 55636, Thessaloniki, Greece; First Propaedeutic Internal Medicine Department, AHEPA University General Hospital of Thessaloniki, 55636, Thessaloniki, Greece; Emergency Department, AHEPA University General Hospital of Thessaloniki, 55636, Thessaloniki, Greece

**Keywords:** patient triage, Artificial Intelligence

## Abstract

**Purpose:**

In the Emergency Departments (ED) the current triage systems that are been implemented are based completely on medical education and the perception of each health professional who is in charge. On the other hand, cutting-edge technology, Artificial Intelligence (AI) can be incorporated into healthcare systems, supporting the healthcare professionals’ decisions, and augmenting the performance of triage systems. The aim of the study is to investigate the efficiency of AI to support triage in ED.

**Patients–Methods:**

The study included 332 patients from whom 23 different variables related to their condition were collected. From the processing of patient data for input variables, it emerged that the average age was 56.4 ± 21.1 years and 50.6% were male. The waiting time had an average of 59.7 ± 56.3 minutes while 3.9% ± 0.1% entered the Intensive Care Unit (ICU). In addition, qualitative variables related to the patient’s history and admission clinics were used. As target variables were taken the days of stay in the hospital, which were on average 1.8 ± 5.9, and the Emergency Severity Index (ESI) for which the following distribution applies: ESI: 1, patients: 2; ESI: 2, patients: 18; ESI: 3, patients: 197; ESI: 4, patients: 73; ESI: 5, patients: 42.

**Results:**

To create an automatic patient screening classifier, a neural network was developed, which was trained based on the data, so that it could predict each patient’s ESI based on input variables.

The classifier achieved an overall accuracy (F1 score) of 72.2% even though there was an imbalance in the classes.

**Conclusions:**

The creation and implementation of an AI model for the automatic prediction of ESI, highlighted the possibility of systems capable of supporting healthcare professionals in the decision-making process. The accuracy of the classifier has not reached satisfactory levels of certainty, however, the performance of similar models can increase sharply with the collection of more data.

## Santrauka.

**Įvadas:**

Skubios pagalbos skyriuose šiuo metu taikomos prioritetų nustatymo sistemos yra visiškai grindžiamos kiekvieno atsakingas pareigas einančio sveikatos apsaugos profesionalo medicininiu išsilavinimu ir subjektyviu situacijos suvokimu. Tačiau, kita vertus, šiuo metu moderniausia technologija – dirbtinis intelektas – gali būti integruota į sveikatos apsaugos sistemą paremti sveikatos apsaugos profesionalų sprendimus ir pagerinti prioritetų nustatymo sistemų veiklos rezultatus. Tyrimo tikslas – ištirti AI efektyvumą paremiant prioritetų nustatymą skubios pagalbos skyriuose.

**Pacientai ir metodai:**

Į šį tyrimą buvo įtraukti 332 pacientai. Surinkti 23 įvairūs rodikliai, nusakantys jų būklę. Apdorojus pacientų duomenis ir gautą informaciją, paaiškėjo, kad jų vidutinis amžius buvo 56,4±21,1 metai ir kad 50,6 % pacientų buvo vyrai. Vidutinis laukimo laikas buvo 59,7±56,3 minutės, o 3,9 %±0,1 % pacientų buvo pasiųsti į intensyvios terapijos skyrių. Be to, pasitelkti kokybiniai kintamieji, susiję su paciento sveikatos istorija ir priėmimu į sveikatos apsaugos įstaigą. Taip pat surinkti šie tiksliniai kintamieji: hospitalizacijos dienos (vidurkis 1,8±5,9) ir skubaus atvejo sunkumo indeksas (ESI), jo pasiskirstymas buvo: ESI: 1–2 pacientai, ESI: 2–18 pacientų, ESI: 3 –197 pacientai, ESI: 4–73 pacientai, ESI: 5–42 pacientai.

**Rezultatai:**

Automatiniam pacientų atrankos klasifikatoriui sudaryti sukurtas neuroninis tinklas, kuris buvo apmokytas remiantis surinktais duomenimis, kad būtų galima prognozuoti kiekvieno paciento ESI, pasitelkiant pradinius įvesties kintamuosius. Klasifikatorius pasiekė bendrąjį 72,2 % tikslumą (F1 rezultatą) net nepaisant to, kad buvo susidurta su klasių disbalansu.

**Išvados:**

Dirbtinio intelekto modelio sukūrimas ir įgyvendinimas siekiant automatiškai prognozuoti ESI vertes atskleidė galimybę kurti sistemas, gebančias padėti sveikatos apsaugos profesionalams priimant sprendimus. Klasifikatoriaus tikslumas dar nėra pasiekęs patenkinamo užtikrintumo lygio, tačiau panašių modelių veiklos rezultatai gali būti labai pagerinti surinkus didesnį duomenų kiekį.

## Introduction

Nowadays an ever-increasing number of patients visit the emergency department (ED), asking for medical advice and care, inducing maximized workload and consequently delay in the provision of medical services, diagnosis, and treatment [1]. It has been reported that such overcrowding in ED entails augmentation of requisite time for medical personnel to take action, leading to increased percentages of morbidity and mortality in a lot of medical conditions [2]. Moreover, this situation affects negatively the patients’ containment and patience, as well as the performance and mental state of ED employees [3]. In the modern healthcare systems, not only in developed countries but also in developing, ED triage is been used widely since the 1950s [4]. The aim of this method is the immediate assortment of patients, according to their clinical condition, in high-urgency and low-urgency patients [5]. This is of critical importance because patients with severe symptoms will be treated as soon as possible, minimizing the negative effects of a long waiting time [4]. Recently, different Triage Scales have been applied, in order to maximize the efficacy of ED’s contribution to public health. Among the most commonly known and used scales are the Australasian Triage Scale (ATS), the Canadian Triage and Acuity Scale (CTAS), the Emergency Severity Index (ESI), and the Manchester Triage System (MTS) [5,6]. It is easily understood, that contemporary problems require innovative and cutting-edge solutions, rendering Artificial Intelligence (AI) to one of them [7]. AI is a set of mathematical and logical algorithms, aiming to simulate human intelligence, cognition, and expertise, processed by machines, namely computer systems [8]. Based on relevant medical applications, the application of AI to ED shows promise, when implemented for the classification of patients, diagnoses, and treatment which will have the potential to be conducted as fast and efficiently as possible [9]. However, the development of AI models and systems remains challenging, as many burdens can occur during the collection and processing of data and its outcomes cannot always be interpreted [7,10]. The purpose of this study is to underline the fact that the implementation of AI can improve conspicuously the quality and effectiveness of health care systems, making these novel techniques necessary.

## Methods

Data were thoroughly collected from 332 patients that were brought or came willingly to the Emergency Department of AHEPA University Hospital during the months of October-December of 2021. Throughout the process of data collection, specific inclusive and exclusive criteria were defined. Concretely, in the study were included patients who were above 16 years old and who were neither COVID-19 infected nor suspected.

A total number of 23 variables, both numerical and categorical, all related to the patients’ conditions were gathered for each patient. Initial analysis of the collected dataset showed that the average age was 56.4 ± 21.1 years, 50.6% were male. The waiting time had an average of 59.7 ± 56.3 minutes while 3.9% ± 0.1% entered the ICU. In addition, qualitative variables related to the patient’s history and admission clinics were used.

Two potential target variables were selected, namely the days of stay in the hospital, which were by average 1.8 ± 5.9, and the Emergency Severity Index (ESI) for which the following distribution applies: ESI: 1, patients: 2; ESI: 2, patients: 18; ESI: 3, patients: 197; ESI: 4, patients: 73; ESI: 5, patients: 42. Following an investigation of the feasibility and value of predicting the potential targets, the hospitalization days were excluded and the ESI variable was selected.

Given the nature of the problem, for the development of an automatic patient screening classifier, an artificial neural network (ANN) architecture was selected, namely a feedforward neural network [11]. The network was trained on 80% of the total number of patients and used as inputs all variables except the ESI which, as mentioned above, was selected as the output target. The data was randomized to avoid potential biases and were tested and evaluated repeatedly to ensure the generalization of the outcomes.

## Results

The ANN was trained for an average of 21 epochs considering all runs. Several metrics were considered for evaluating the performance of the trained model, including accuracy, precision, recall, and F1 score. Since the target classes were unbalanced, F1 score was primarily selected as the desired performance metric. The produced classifier managed to achieve an overall accuracy (F1 score) of 72.2% even though there was an imbalance in the classes. A confusion matrix is used to show the number of predictions over the true labels for each class, i.e. the ESI. This is a helpful tool to visualize the results and identify which class suffers from multiple false predictions, as well as confirm the correct predictions (diagonal, gray cell in Table 1). The confusion matrix and a detailed analysis of the results are presented in Table 1 and Table 2 respectively.

**Table 1.** Confusion matrix of the classification results.

**True ESI**						
**Predicted ESI**
		**1**	**2**	**3**	**4**	**5**
	**1**	2	1	2	1	0
	**2**	0	12	12	2	1
	**3**	0	4	169	4	2
	**4**	0	1	11	63	4
	**5**	0	0	3	3	35

**Table 2.** Performance metrics for each class (ESI).

**Class (ESI)**	**Truth**	**Predictions**	**Accuracy**	**Precision**	**Recall**	**F1 score**
1	2	6	0.99	0.33	1.0	0.50
2	18	27	0.94	0.44	0.67	0.53
3	197	179	0.89	0.94	0.86	0.90
4	73	79	0.92	0.80	0.86	0.83
5	42	41	0.96	0.85	0.83	0.84

The overall accuracy of the classifier reaches 84.6%, however, the F1 score, a more appropriate performance metric in unbalanced datasets, reaches 72.2%.

## Discussion

The implementation of triage systems in health care units is crucial for the regular provision of qualitative medical services [12]. A commonly used triage system, also applied in our hospital, is ESI, which consists of a five-level triage scale and was created in the U.S. by ED physicians Richard Wuerz and David Eitel [13]. ESI is used not only for the definition of the patients who should be examined and treated first, according to their clinical severity but also for the consideration of resources that are about to be used for the patient’s disposition [6,13]. Patients who are potentially dying are assigned to level 1 (immediate treatment), those who should not wait are considered level 2 (emergency treatment), and those who are considered safe to wait are ranked to level 3 (urgent treatment) through 5 (non-urgent) by anticipated resource use [14]. Although ESI facilitates the medical processes, a variety of inaccuracies may occur during its application, due to the fact that this classification is a subjective tool, as different triage medical staff can evaluate the same medical case in a non-identical way [13]. ESI, as each triage system uses clinical signs, including body temperature, heart rate, respiratory rate, oxygen saturation, blood pressure, and work of breathing in order to foresee the illness’s severity and their chief complaints [4,15]. However, this way of evaluation may be misleading, as patients with a dangerous health condition can present normal vital signs [4]. For the aforementioned reasons, it is of utmost importance the implementation of AI in healthcare systems, as it can contribute to decision-making and problem-solving [8].

In order to potentially transform the clinical practice, a variety of algorithms have been tested. The most famous algorithms that have been used and provide critically improved effectiveness in classification tasks are Logistic Regression, Decision Tree, Ensembles, Naïve Bayes, Support Vector Machines, and Artificial Neural Networks [16]. These algorithms are able to create AI models utilize with large amounts of data to self-improving their prediction accuracy, by a method called training [17]. The inputs, and preferable the output labels, that are used for training the models, must be derived from the statistical analysis of information that has been collected in the healthcare unit [7,17].

In our case, an artificial neural network, namely a perceptron, has been trained and tested based on the inputs of patients, such as vital signs, main complaint, personal history, etc., in order to automatically predict the ESI level. It must be underlined that majority of the patients were sorted in level 3 of ESI, whereas only 2 patients were in level 1. The accuracy (F1 score) of the classifier (72.2%) indicates the impact of AI on the improvement of ED triage.

Although decision support systems (DSS) based on AI models can be beneficial in the patient management and enhance ED triage, they exhibit a variety of limitations and burdens [3,18]. Specifically, overstated risk aversion hinders automated triage systems to have a central point on safety and these systems offer patients advice that is diagnostic inaccurate and causes the needlessly search for physical care by them [3]. Another troublesome requirement is that in order to achieve high prediction accuracy, ergo maximum effectiveness, AI models require large amounts of curated and labeled patient data for training [17]. This ensures that, under all circumstances, they will be able to handle the complexity of comorbidities that are frequently seen in the population [19,20]. It is essential for the future welfare of healthcare systems, and medical professionals to evaluate the patients’ condition not only from the results of the digital triage but also from the patients’ clinical condition. Following this process, technology should be implemented supplementary and not as the only tool for patient management.

The limitations of our study can be attributed to the tenor of AI. Specifically, it is a necessity for the optimal application of AI, the collection and processing of data and its outcomes to be carefully applied. So, we acknowledge that this classifier based on AI has still opportunities for improvements, in order that all the questions raised be answered. Moreover, it is important to comprehend that using artificial intelligence based techniques will not undoubtedly result in classification or prediction that is more effective than the already existing human systems, but the role of AI is supportive as we already mentioned.

## Conclusion

The application of AI in support of triage systems in ED can be undoubtedly an auxiliary tool that can sharply improve the effectiveness of healthcare units. With the automatic prediction of ESI, even though the classifier has not reached satisfactory levels of certainty yet, issues that currently preoccupy the healthcare professionals could be diminished or even eradicated. However, the performance of similar models can intensely increase with the collection of more data.
